# Determinants of hypertensive crisis among hypertensive patients at adult emergency departments of public hospitals in Addis Ababa, Ethiopia, 2021: a case–control study

**DOI:** 10.1186/s12245-023-00549-2

**Published:** 2023-10-09

**Authors:** Hailemariam Gezie, Aklilu Azazh, Birhanu Melaku, Habtam Gelaye

**Affiliations:** 1 Department of Emergency and Critical Care Nursing, School of Nursing and Midwifery, College of Medicine and Health Sciences, Wollo University, Dessie, Ethiopia; 2 Department of Emergency Medicine, School of Medicine, College of Health Sciences, Addis Ababa University, Addis Ababa, Ethiopia; 3 Department of Psychiatry, School of Nursing and Midwifery, College of Medicine and Health Sciences, Wollo University, Dessie, Ethiopia

**Keywords:** Hypertension, Hypertensive crisis, Public hospitals

## Abstract

**Background:**

Hypertension (HTN) is a major global health problem that affects approximately 1.13 billion people worldwide, and 1–2% of this population has hypertensive crisis. Hypertensive crisis is becoming a major health issue in low-income countries. However, few studies have been conducted in developing countries such as Ethiopia. This study aimed to assess the determinants of hypertensive crisis among patients visiting adult emergency departments of public hospitals in Addis Ababa.

**Method:**

A hospital-based unmatched case–control study was conducted among 85 cases with a hypertensive crisis and 170 controls with hypertension without a hypertensive crisis in the adult emergency departments of public hospitals in Addis Ababa from March 15 to May 15, 2021. Data were collected using a structured questionnaire and analyzed using SPSS version 26. Binary logistic regression and multivariable logistic regression were performed. Finally, a statistically significant level was declared at a *p* value of less than 0.05. The result was summarized and presented in text, tables, and graph.

**Result:**

The odds of having hypertensive crisis were 3.6 times (AOR = 3.621) higher among participants with a history of hypertension compared to those without a history of hypertension. There was also 4 times increased risk of hypertensive crisis among participants who presented with diabetes mellitus than participants who presented without it (AOR = 4.179). Similarly, participants who presented with stroke had 7 times higher odds of having hypertensive crisis (AOR = 7.174) than participants without stroke.

**Conclusion:**

This study demonstrated a statistically significant association between unemployment, diabetes mellitus, stroke, heart failure, history of hypertension, family history of hypertension, and regular follow-up with a hypertensive crisis. The Ethiopian Ministry of Health, Ababa City Administration Health Bureau, and hospitals shall give due attention to the HTN crisis. Health care workers, hospital managers, and other stakeholders shall work towards the early detection and management of HTN-crisis to prevent related morbidity, disability, and mortality.

## Introduction

Hypertension (HTN) is a major global health problem that affects approximately 1.13 billion people worldwide. Approximately 7.1 million annual deaths due to HTN are recorded worldwide [1–4]. HTN is mainly characterized by increased arterial blood pressure and is the major contributing factor to morbidity and mortality in both low-income and high-income countries [5]. In Africa, its prevalence is higher than in other continents, specifically in the sub-Saharan region [6, 7]. A systematic review and meta-analysis shows the pooled prevalence of HTN in Ethiopia is 20.63% [8]. In Addis Ababa, its prevalence is also high (29.24%) [9].

Hypertensive crisis (HTN-crisis) is an acute, severe rise in BP that can be diagnosed in patients with BP ≥ 180/120 mmHg [10, 11]. There are two types of HTN crisis. The first is HTN emergency, which was first described by Volhard and Fahr in 1914 [12] and denotes BP ≥ 180/120 mmHg with evidence of serious vital organ damage, mostly in the heart, brain, kidneys, eyes, lungs, and blood vessels, necessitating rapid diagnosis and appropriate management to reduce or avoid end-organ damage. The HTN emergency can also be explained based on the organs involved in aortic dissection, acute hypertensive pulmonary edema, acute myocardial infarction, acute renal failure, hypertensive encephalopathy, acute coronary syndrome, focal neurologic deficits, stroke, and hypertensive retinopathy [13–15]. Its consequences on different organs account for 36% of cardiovascular conditions, including acute heart failure, 24% of cerebral infarction, 16% of hypertensive encephalopathy, 12% of acute coronary syndrome (acute myocardial infarction and/or unstable angina), 4.5% of eclampsia during pregnancy, 4% of intracerebral or subarachnoid hemorrhage, and 2% of aortic dissection [16]. The second is HTN-urgency, which can be defined as a severe acute increase in BP without evidence of acute organ damage [4, 15, 17, 18].

Hypertensive crisis accounts for 1–2% of the global HTN prevalence [2, 12, 19]. According to literature, HTN crisis accounts for 1–15% of known hypertensive patients who had been treated previously [12, 20, 21]. It can cause significant morbidity and mortality worldwide [22]. Approximately 25% of all medical emergency department (ED) visits were due to HTN emergency [1]. The HTN emergency-related annual death rate is > 79%, with a median 10.4-month length of survival if not managed properly [10].

Although the HTN crisis is the reason for increased morbidity, mortality, and disability, it is still an underestimated problem in low- and middle-income countries, including Ethiopia. However, very few studies have been conducted in sub-Saharan Africa and no research has been conducted on its determinants in Ethiopia. Therefore, this study aimed to identify the determinant factors of HTN-crisis among adult patients at the ED of public hospitals in Addis Ababa, Ethiopia.

## Methods and materials

### Study setting and period

The study was conducted at the adult ED of seven selected public hospitals in Addis Ababa, including the country’s largest hospitals (namely: Tikur Anbessa Specialized Hospital (TASH), Saint Paul’s Hospital Millennium Medical College (SPHMMC), Saint Peter’s Specialized Hospital (SPSH), Addis Ababa Burn Emergency & Trauma hospital (AaBET), Yekatit 12 Specialized Hospital (Y-12SH), Menelik II Specialized Hospital (M-IISH), and Tirunesh Bejing Hospital (TBH)). These hospitals serve all patients across the country. The study hospitals were selected using the lottery method from 15 public hospitals in the capital. This study was conducted from March 15 to May 15, 2021.

### Study design

A hospital-based unmatched case–control study was conducted.

### Population

#### Source population

All adult patients with HTN who visited the ED of public hospitals in Addis Ababa were the source population.

#### Study population

All adult patients with HTN, who visited the ED of the selected public hospitals in Addis Ababa from the 15th of March to the 15th of May 2021, were the study population.

### *Eligibility* criteria

#### Inclusion criteria

All adult patients who visited the ED of the selected hospitals during the study period with HTN crisis were included as cases.

All selected adult patients who visited the ED of the selected hospitals during the study period with HTN without HTN crisis were included as controls. The controls were presented to the ED of the hospitals for the management of other disease conditions.

#### ***Exclusion criteria***

- All HTN patients’ age is below 18 years.
- Patients who fulfilled the inclusion criteria but were not voluntary to participate in the study.
- Patients who fulfilled the inclusion criteria but were not mentally competent.
- Patients who fulfilled the inclusion criteria but were seriously ill and unresponsive during the study period

### Sample* size determination*

To determine the sample size, various factors significantly associated with the outcome variable were considered in a previous study and a larger sample size was taken for this study. The required sample size was determined using Epi info version 7.0 and the double population proportion formula with the assumptions of 95% CI, 80% power, a case-to-control ratio of 1:2, and a 5% margin of error. The percentage of controls exposed (P2) and the corresponding adjusted odds ratio were taken as 63.6% and 2.494, respectively, from a previous study [23]. The total sample size was 252 (84 cases and 168 controls). For the possibility of non-respondents, 5% of the total sample size was added and the final sample comprised 265 participants (88 cases and 177 controls).

### Sampling* technique*

Based on the 2019 reports obtained from the ED registry and Electronic Health Management Information System of the study hospitals, 116 patients with HTN-crisis visited the ED from March 15 to May 15, 2019. Taking these data as a baseline, an estimated number of study subjects was allocated proportionally to the selected hospitals, as shown in Table 1. All eligible cases were included using a consecutive sampling technique and controls were selected using simple random sampling per case until the calculated sample size was obtained.

**Table 1:** Sampling technique and proportional allocation of sample to assess determinants of HTN-crisis among patients at adult emergency departments of hospitals in Addis Ababa, Ethiopia, 2021 (*N* = 255, P1 = 85, P2 = 170)

Serial number	Name of selected hospitals	2019 Case flow over 2 months (March 15 to May 15)	Proportionally allocated sample
1	TASH	20	15
2	SPHMMC	19	14
3	SPSH	22	17
4	AaBET	10	7
5	Y-12 SH	10	8
6	M-II SH	14	11
7	TBH	21	16
Total		116	88

### Data collection tools

Data were collected using a structured questionnaire adapted from previous studies [3, 23–29]. The tools comprised six parts: sociodemographic variables, patients’ vital signs (BP), comorbidities, and behavioral and other determinants. Participants’ adherence to their antihypertensive medications was assessed using an 8-item Morisky medication adherence assessment tool adapted from a previous study [30, 31]. Participants who scored less than 6 out of 8 were considered to have a low adherence level. Those scoring above 6 but below 8 were also considered to have a medium adherence level and those who scored 8 were considered to have a high adherence level. Participants’ knowledge of hypertension was also assessed using a 14-item assessment tool adapted from a previously conducted study [32]. Participants who scored below the mean were considered to have poor knowledge and those who scored the mean and above were considered to have good knowledge. The questionnaire was translated into Amharic during data collection for interviews and back to English during analysis.

### Data collection procedures

Seven nurses as data collectors, one MSc nurse as a supervisor, and one data clerk were recruited and trained on the objectives of the study, nature of the variables, approach to the participants, and other issues. Data were collected through face-to-face interviews with respondents using structured and pre-tested questionnaires. The same interview was conducted for both the cases and controls. Data regarding the patients’ diagnoses, vital signs, and comorbidities were obtained from their medical records.

### Data quality assurance

The questionnaire was checked for coherence and completeness and pretested among 5% of the total sample size at Zewditu Memorial Hospital located in Addis Ababa. One-day training was given to data collectors, the data clerk, and the supervisor prior to the actual data collection period. The collected data were checked daily for completeness and appropriateness. The internal consistency of the questionnaire was checked using Cronbach’s alpha, with a score of 0.84.

### Data analysis and presentation

The collected data were coded and entered into Epi data version 4.6 and then exported to SPSS version 26 for analysis. Descriptive statistics were used to determine the distribution of variables and were presented using text, frequency tables, and graphs. Pearson’s chi-square test was performed to assess the association between the variables and HTN crisis. Binary logistic regression was also performed for each variable and variables with a *p* value less than 0.25 were eligible for the final model. Multivariable logistic regression was performed to identify the independent predictors of HTN crisis and to control the effect of potential confounding variables using adjusted odds ratios (AOR) with corresponding 95% confidence intervals. Model fitness was also checked using the Hosmer–Lemeshow fitness test. Finally, variables with a *p* value less than 0.05 were declared as statistically significant.

## Result

In this hospital-based unmatched case–control study, 255 participants (85 cases and 170 controls) who visited the seven selected public hospitals in Addis Ababa [TASH = 15, SPHMMC = 14, SPSH = 16, AaBET = 7, Y-12SH = 8, M-IISH = 10, and TBH = 15] were involved with 96.23% response rate.

### Sociodemographic characteristics of participants

The mean age of the participants was 56.15 ± 14.004 for cases and 55.34 ± 12.768 for controls. 54.1% of cases and 57.6% of controls were under the age group of 45–65 years. The proportion of males was higher than females in both the cases (57.6%) and controls 69.4%). Similarly, the proportion of urban residents was higher among cases and controls with the proportion of 70.6% and 82.9%, respectively. Regarding the employment and marital status of the participants, a higher proportion of cases and controls were employed and married (63.5% vs. 78.8% and 50.6% vs. 68.2%, respectively) (Table 2).

**Table 2:** Sociodemographic characteristics of participants at public hospitals in Addis Ababa, Ethiopia, 2021 (*N* = 255, P1 = 85, P2 = 170)

Variables		Group of patients (*N* = 255)		*X*^2^, *P* value
		Cases, *n* (%)	Controls, *n* (%)	
**Age category**	18–45	17 (20.0)	36 (21.2)	*X*^2^ = 0.714, *P* > 0.05
	46–65	46 (54.1)	98 (57.6)	
	> 65	22 (25.9)	36 (21.2)	
**Sex**	Female	36 (42.4)	52 (30.6)	*X*^2^ = 3.470, *P* > 0.05
	Male	49 (57.6)	118 (69.4)	
**Residence**	Rural	25 (29.4)	29 (17.1)	*X*^2^ = 5.180, *P* < 0.05
	Urban	60 (70.6)	141 (82.9)	
**Educational status**	None educated	15 (17.6)	25 (14.7)	*X*^2^ = 3.993, *P* > 0.05
	Elementary school	24 (28.26)	35 (20.6)	
	Secondary school	12 (14.1)	39 (22.9)	
	Tertiary	34 (40.0)	71 (41.8)	
**Employment**	Unemployed	31 (36.5)	36 (21.2)	*X*^2^ = 6.843, *P* < 0.01
	employed	54 (63.5)	134 (78.8)	
**Marital status**	Married	43 (50.6)	116 (68.2)	*X*^2^ = 13.562, *P* < 0.01
	Single	29 (34.1)	25 (14.7)	
	Divorced	6 (7.1)	17 (10.0)	
	widowed	7 (8.2)	12 (7.1)	
**Monthly income in ETB**	< 500	7 (8.2)	17 (10.0)	*X*^2^ = 0.928, *P* > 0.05
	500–999	14 (16.5)	21 (12.4)	
	≥ 1000	64 (75.3)	132 (77.6)	

Key: *n* = number of participants, ETB Ethiopian Birr, *X*^2^ = chi-square

The mean SBP with its standard deviation was 195.06 ± 18.989 for cases and 151.98 ± 16.489 for controls. The mean DBP was also 116.19 ± 13.514 for cases and 97.42 ± 9.751 for controls.

### Participants’ comorbid conditions

**Fig. 1:**
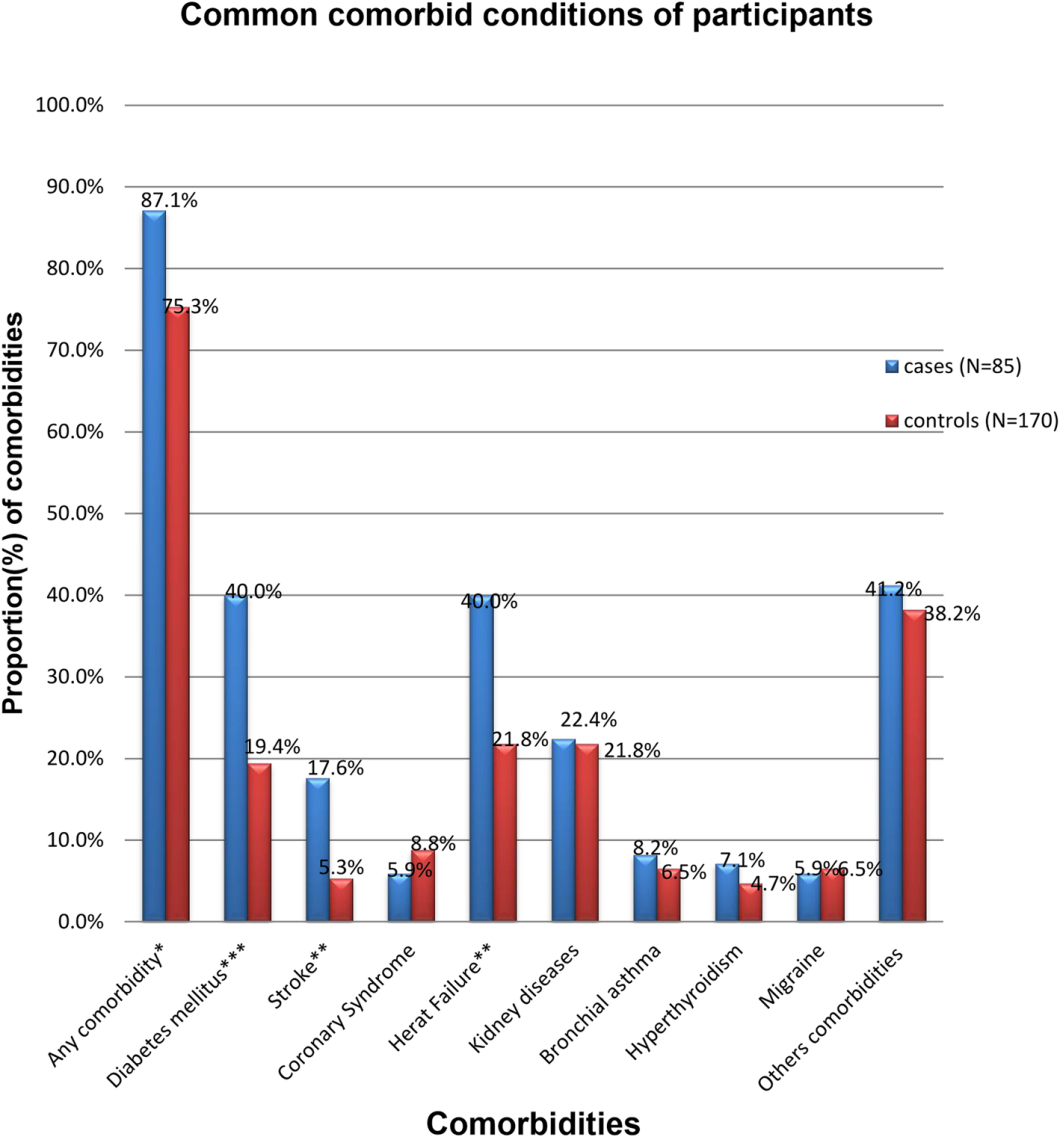
Comorbid determinants of HTN crisis among patients at adult emergency departments of hospitals in Addis Ababa, Ethiopia, 2021 (*N* = 255, P1 = 85, P2 = 170)

Most participants in both groups presented with different comorbidities (87.1% of cases and 75.3% of controls). The proportion of participants who presented with DM and heart failure was equally higher among cases than among controls (40%), followed by those with kidney disease (22.4%). In contrast, the proportion of other comorbidities was 41.2% and 38.2% in the cases and controls, respectively (Fig. 1).

### Behavioral and other determinants

Most participants in the case and control groups had a history of HTN (82.4% and 64.1%, respectively). Approximately 67.1% of the cases and 61.8% of the controls were taking antihypertensive medications. Similarly, only 18.8% of cases and 35.3% of controls had regular follow-up. Approximately 72.9% of cases and 57.1% of controls had a family history of hypertension. The proportion of cigarette smokers was slightly higher among cases than among controls (21.2% and 15.9%, respectively). Similarly, the proportion of participants who drank alcohol among the cases (41.2%) was higher than that of the controls (32.4%). On the other hand, the proportion of participants who performed physical exercise among the cases (25.9%) was less than that of the controls (28.8%) (Table 3).

**Table 3:** Behavioral determinants of HTN crisis among patients at adult emergency departments of public hospitals in Addis Ababa, Ethiopia, 2021 (*N* = 255, P1 = 85, P2 = 170)

Variables			Group of patients		***X***^2^, ***P*** value
			Cases, *n* (%)	Controls, *n* (%)	
History of previous HTN			70 (82.4)	109 (64.1)	*X*^2^ = 9.007, *P* < 0.01
Regular follow-up			16 (18.8)	60 (35.3)	*X*^2^ = 7.348, *P* < 0.01
Family history of HTN			62 (72.9)	97 (57.1)	*X*^2^ = 6.089, *P* < 0.05
Smoking cigarettes (*N* = 255)			18 (21.2)	27 (15.9)	*X*^2^ = 1.093, *p* > 0.05
Average daily consumption of cigarettes in pieces		≥ 20	7 (38.9)	10 (37.0)	*X*^2^ = 0.016, *P* > 0.05
		< 20	11 (61.1)	17 (63.0)	
Alcohol drinking (*N* = 25)			35 (41.2)	55 (32.4)	*X*^2^ = 1.932, *p* > 0.05
Type of alcohol	Beer		17 (48.6)	30 (55.6)	*X*^2^ = 0.416, *P* > 0.05
	Wine		5 (14.3)	10 (18.5)	*X*^2^ = 0.272, *P* > 0.05
	Traditional alcoholic drinks		23 (65.7)	39 (72.2)	*X*^2^ = 0.426, *P* > 0.05
	Other alcoholic drinks		8 (23.5)	13 (24.1)	*X*^2^ = 0.003, *P* > 0.05
Regular physical exercise (*N* = 25)			22 (25.9)	49 (28.8)	*X*^2^ = 0.244, *p* > 0.05
Frequency of exercise per week in days		< 3 days	13 (59.1)	30 (61.2)	*X*^2^ = 0.029, *P* > 0.05
		≥ 3 days	9 (40.9)	19 (38.8)	
Type of exercise		Walking	17 (77.3)	39 (79.6)	*X*^2^ = 0.049, *P* > 0.05
		Jumping	5 (22.7)	11 (22.4)	*X*^2^ = 0.001, *P* > 0.05
		Other exercises	10 (45.5)	20 (40.8)	*X*^2^ = 0.134, *P* > 0.05

Key: *X*^2^ = chi-square, *n* = number of participants, *N* = sample size

**Other alcoholic drinks** include those other than the above list available with different names

**Other exercises** include running and any other physical activity including gymnasium

In this study, approximately equal proportions of both groups (47.4% of cases and 46.7% of controls) had a medium level of adherence to antihypertensive medications. Similarly, approximately equal proportions of both groups had a low adherence level. On the other hand, only 21.0% of cases and 21.9% of controls had a high level of adherence. This study found that a higher proportion of both cases and controls (70.6% and 71.2%, respectively) had good knowledge.

### Factors associated with hypertensive crisis

Explanatory variables with a *p* value < 0.25 in the binary logistic regression were eligible for multivariable logistic regression analysis. The explanatory variables were sex, address, employment, marital status, presence of any comorbidity, diabetes mellitus, stroke, heart failure, history of HTN, regular follow-up, family history of HTN, and alcohol consumption. After multivariable logistic regression and adjustment were performed, there was a significant association of seven variables with the HTN crisis.

DM was a statistically significant determinant factor for HTN-crisis in which there were 4 times higher odds of having HTN crisis among participants with DM (AOR = 4.064; 95% CI 1.86, 8.87) than among those without DM.

Stroke was also a statistically significant determinant factor for HTN-crisis in which the odds of having HTN crisis were 7 times higher among participants with stroke than among those without stroke (AOR = 7.174; 95% CI 2.29, 22.44).

On the other hand, there was a statistically significant association of having regular follow-ups and HTN crisis in which participants who had regular follow-ups were less likely to develop HTN crisis than those who did not undergo regular follow-up (AOR = 0.222; 95% CI 0.10, 0.51) (Table 4).

**Table 4:** Factors associated with HTN crisis among patients at adult emergency departments of public hospitals in Addis Ababa, Ethiopia, 2021 (*N* = 255, P1 = 85, P2 = 170)

Variable name	**COR (95% CI)**	**AOR (95% CI)**
Sex (female)	1.667 (0.97, 2.86)	1.432 (0.73, 2.82)
Residence (rural)	2.026 (1.10, 3.75)	1.985 (0.94, 4.19)
Marital status (single)	3.129 (1.65, 5.93)	2.135 (0.97, 4.71)
Unemployment	2.137 (1.20, 3.80)	2.974 (1.38, 6.42) ^**^
Comorbidity	2.207 (1.07, 4.55)	0.889 (0.33, 2.40)
DM	2.768 (1.56, 4.93)	4.064 (1.86, 8.87) ^***^
Stroke	4.990 (1.95, 12.77)	7.174 (2.29, 22.44) ^**^
Heart failure	2.369 (1.36, 4.22)	2.911 (1.35, 6.26) ^**^
History of HTN	2.612 (1.38, 4.95)	3.621 (1.63, 8.02) ^**^
Regular follow up	0.245 (0.23, 0.80)	0.222 (0.10, 0.51) ^***^
Family history of HTN	2.029 (1.15, 3.58)	2.263 (1.12, 4.58) ^*^
Drinking alcohol	1.464 (0.85, 2.51)	1.270 (0.65, 2.48)

Key: *p < 0.05, **p value < 0.01, ***p < 0.001, *COR* crude odds ratio, *AOR* adjusted odds ratio, *CI* confidence interval

## Discussion

The result of this study indicated that there were 3 times higher risk of having HTN-crisis among unemployed participants than among those employed (AOR = 2.974; 95% CI 1.38, 6.42). This result was supported by a study done in European member countries which approved unemployment as a contributing risk factor for many cardiovascular diseases including hypertensive crisis [33]. One possible reason might be that being unemployed can cause stress, which further causes and aggravates the HTN crisis. The other reason might be that unemployed people also cannot get enough money for health care bills and in these people, especially those with chronic conditions, their status may worsen.

In this study, there were 4 times higher odds of having HTN crisis among participants with DM (AOR = 4.064; 95% CI 1.86, 8.87) than among those without DM. This result was supported by a longitudinal study conducted in Bahrain [34], a retrospective study conducted in Brazil [35], and a case–control study conducted in the USA [25]. This consistency to the USA study might be due to the similarities in the study designs, and the study participants in both studies were adults. The consistency of the Bahrain and Brail studies might be the similarities in the age group of participants which is above 18 years in all studies. The other reason for illnesses with the three studies might be the blood vessel wall endothelium of diabetes patients has a reduced synthesis of vasodilators and an increased release of pro-coagulants and vasoconstrictors, which lead to atherosclerosis and further results in high blood pressure [36]. However, this significance was not supported by another matched case–control study conducted in the USA, which found no association between DM and HTN crisis [28]. This discrepancy might be due to better health care access to people in the USA and better patient adherence to preventive as well as treatment modalities. The economic statuses of the people in the two countries are also different.

This study also revealed that the odds of having HTN crisis were 7 times higher among participants with stroke than among those without stroke (AOR = 7.174; 95% CI 2.29, 22.44). This significance was not supported by a cross-sectional study conducted in Uganda, which did not reveal any association between stroke and HTN crisis [26]. This discrepancy might be due to variations in the study design and sample size. However, this association was supported by a case–control study conducted in the USA [25]. This might be due to the similarity in study design.

There were 3 times higher odds of having HTN-crisis among participants with heart failure compared to participants without heart failure (AOR = 2.911; 95% CI 1.35, 6.26). This result was in line with a longitudinal study conducted in Switzerland and two case–control studies conducted in the USA [25, 28]. This similarity with the Switzerland study might be due to the similarity of the source of data (both this study and the Switzerland study used prospective data) and the similarity in study design with the two case–control studies conducted in the USA.

The study also showed 3.6 times higher odds of having HTN-crisis among participants with a history of HTN compared to those without a history of HTN (AOR = 3.621; 95% CI 1.63, 8.04) which was supported by a study conducted in Indonesia [21]. This result contradicted the cross-sectional studies conducted in Ethiopia and Uganda [23, 26]. This difference might be because these were retrospective cross-sectional descriptive studies that were not conducted to assess the determinant factors, whereas this study was conducted prospectively to assess the determinant factors of the HTN crisis.

This study also revealed that participants who had regular follow-ups were less likely to develop HTN crisis than those who did not undergo regular follow-ups (AOR = 0.222; 95% CI 0.10, 0.51). It is believed that those people who had regular follow-ups for their HTN crisis could control their BP better than those who had no follow-up and this study proves this assumption. When people have regular follow-ups, they can know the trend of their BP measurement and they can obtain updated information regarding their condition, their treatment, and the occurrence of complications.

According to this study’s finding, there were 2.26 times higher odds of having HTN crisis among participants with a family history of HTN than among participants without the condition (AOR = 2.263; 95% CI 1.12, 4.58). This might be due to the genetic predisposition to the HTN-crisis and those participants with a family history of HTN might also share similar socio-cultural and living environments with their families, which can be a risk factor for the HTN crisis.

## Conclusion and recommendations

This study demonstrated an increased odds of having HTN crisis among participants with unemployment, DM, stroke, heart failure, history of hypertension, and family history of hypertension compared to those participants without these conditions. On the other hand, participants who had regular follow-ups were less likely to develop HTN crisis. The Ethiopian Ministry of Health, Ababa City Administration Health Bureau, and hospitals shall give due attention to HTN crisis. Health care workers, hospital managers, and other stakeholders shall work towards the early detection and management of the HTN crisis to prevent related morbidity, disability, and mortality.

## Data Availability

All data supporting the findings of this study are presented in the manuscript. Additional details and raw data are presented with the corresponding author for reasonable requests.

## Abbreviations

AaBET: Addis Ababa Burn Emergency & Trauma Hospital
BP: Blood pressure
CVD: Cardiovascular disease
DM: Diabetes mellitus
ED: Emergency department
HTN: Hypertension
M-IISH: Menelik-II Specialized Hospital
SBP: Systolic blood pressure
SPHMMC: Saint Paul’s Hospital Millennium Medical College
SPSH: Saint Peter’s Specialized Hospital
SPSS: Statistical Package for Social Sciences
TASH: Tikur Anbessa Specialized Hospital
TBH: Tirunesh Bejing Hospital
USA: United States of America
Y-12SH: Yekatit-12 Specialized Hospital
